# Near-Infrared Spectroscopy Assessments of Regional Cerebral Oxygen Saturation for the Prediction of Clinical Outcomes in Patients With Cardiac Arrest: A Review of Clinical Impact, Evolution, and Future Directions

**DOI:** 10.3389/fmed.2020.587930

**Published:** 2020-10-29

**Authors:** Ryosuke Takegawa, Kei Hayashida, Daniel M. Rolston, Timmy Li, Santiago J. Miyara, Mitsuo Ohnishi, Tadahiko Shiozaki, Lance B. Becker

**Affiliations:** ^1^Laboratory for Critical Care Physiology, Feinstein Institutes for Medical Research, Northwell Health System, Manhasset, NY, United States; ^2^Department of Emergency Medicine, North Shore University Hospital, Northwell Health System, Manhasset, NY, United States; ^3^Department of Traumatology and Acute Critical Medicine, Osaka University Graduate School of Medicine, Osaka, Japan; ^4^Department of Emergency Medicine, Donald and Barbara Zucker School of Medicine at Hofstra/Northwell, Manhasset, NY, United States; ^5^Department of Surgery, Donald and Barbara Zucker School of Medicine at Hofstra/Northwell, Manhasset, NY, United States; ^6^Elmezzi Graduate School of Molecular Medicine, Manhasset, NY, United States; ^7^Department of Acute Medicine and Critical Care Medical Center, Osaka National Hospital, National Hospital Organization, Osaka, Japan

**Keywords:** cardiac arrest, cardiopulmonary resuscitation, near-infrared spectroscopy, cerebral oxygen saturation, brain oximetry, ROSC, neurological outcome, prognostication

## Abstract

Despite three decades of advancements in cardiopulmonary resuscitation (CPR) methods and post-resuscitation care, neurological prognosis remains poor among survivors of out-of-hospital cardiac arrest, and there are no reliable methods for predicting neurological outcomes in patients with cardiac arrest (CA). Adopting more effective methods of neurological monitoring may aid in improving neurological outcomes and optimizing therapeutic interventions for each patient. In the present review, we summarize the development, evolution, and potential application of near-infrared spectroscopy (NIRS) in adults with CA, highlighting the clinical relevance of NIRS brain monitoring as a predictive tool in both pre-hospital and in-hospital settings. Several clinical studies have reported an association between various NIRS oximetry measurements and CA outcomes, suggesting that NIRS monitoring can be integrated into standardized CPR protocols, which may improve outcomes among patients with CA. However, no studies have established acceptable regional cerebral oxygen saturation cut-off values for differentiating patient groups based on return of spontaneous circulation status and neurological outcomes. Furthermore, the point at which resuscitation efforts can be considered futile remains to be determined. Further large-scale randomized controlled trials are required to evaluate the impact of NIRS monitoring on survival and neurological recovery following CA.

## Background

Out-of-hospital cardiac arrest (OHCA) remains a major public health challenge worldwide. The global report on OHCA has described that the estimated incidence of OHCA treated *via* emergency medical services (EMS) was 47.3, 40.6, 45.9, and 51.1 per 100,000 person-years in North America, Europe, Asia, and Australia, respectively (i.e., ~4 million cases each year) (1). Despite advances in treatment, such as routine application of targeted temperature management (TTM), neurological prognosis remains poor among survivors of OHCA (2), and there are no reliable methods for predicting neurological outcomes in patients with cardiac arrest (CA) and post-cardiac arrest syndrome (PCAS). International guidelines recommend a multimodal approach for determining prognosis. However, these guidelines are neither universally accepted nor universally implemented, and prognostication may be delayed up to 72 h after restoration of normothermia (3–5).

Physiologically, a prolonged “no-flow” interval during CA followed by low cerebral perfusion during resuscitative management (i.e., “low-flow” status) leads to hypoxic ischemia–reperfusion brain injury—the primary cause of disability after successful resuscitation (6). Near-infrared spectroscopy (NIRS) can be used to obtain continuous, non-invasive measurements of regional cerebral oxygen saturation (rSO_2_) in real time, which may aid in monitoring oxygen metabolism in the brain during this ischemia–reperfusion process. Measurements of rSO_2_ are considered to reflect the balance between cerebral oxygen delivery and consumption in the area of the brain located beneath the device (7). NIRS has been applied in patients with circulatory shock (8), acute brain injury (9, 10), those undergoing perioperative cardiac surgery (11, 12) or carotid endarterectomy (13–15), and during veno-arterial extracorporeal membrane oxygenation in the intensive care unit (ICU) (16–18). Several recent studies have highlighted the feasibility of NIRS for brain monitoring during cardiopulmonary resuscitation (CPR) and after the return of spontaneous circulation (ROSC) in patients with CA (19–24). There are two theoretical uses for NIRS brain monitoring and it is important to understand both. Application of NIRS monitoring may aid in predicting patient outcomes, which may in turn aid clinicians in determining whether to continue or halt resuscitation efforts based on the patient's chance of survival. Alternatively, NIRS measurements may aid in determining the most appropriate resuscitation therapies. For example, patients with low initial NIRS values may benefit from more aggressive resuscitation efforts (e.g., improved CPR, pharmacological treatment, circulatory support). Unfortunately, no previous studies have validated the use of NIRS for either of these purposes. Furthermore, the cut-off rSO_2_ value for predicting good vs. poor clinical outcomes in patients with CA remains to be determined. Publicly available studies have also varied with regard to the timing of NIRS (during CPR or post-ROSC), the clinical setting (prehospital, emergency department [ED], or ICU), and the types of NIRS readings analyzed (initial, mean, highest, or changes in rSO_2_ values over the course of CPR or the ICU stay). Thus, further studies are required to determine the predictive value of NIRS monitoring and its potential for guiding treatment strategies in patients with OHCA (25). In the present review, we discuss the development and evolution of NIRS technology, as well as the potential usefulness of rSO_2_ during CA and post-resuscitation care.

## A Brief Review of NIRS Technology

In 1977, Jöbsis provided the first evidence that NIRS can be used to monitor tissue metabolism *in vivo* (26). Notably, they intended to develop an optical technique for measuring *in vivo* redox changes in the mitochondrial enzyme cytochrome c oxidase (27). They discovered that near-infrared light penetrates deeper into tissues due to its higher tissue transparency, enabling real-time monitoring of changes in the concentrations of light-absorbing molecules within the tissue. Given that hemoglobin chromophores are present in higher concentrations than cytochrome c oxidase, numerous studies have focused on the use of NIRS to measure levels of oxygenated, deoxygenated (or redox/“reduced”), and total hemoglobin (28). In 1985, Ferrari et al. utilized NIRS for continuous, non-invasive monitoring of the human brain (29). In 1995, Müllner et al. provided the first preliminary report regarding the use of NIRS in patients with OHCA, demonstrating that higher median rSO_2_ values during continuous CPR in the ED were associated with better 1-week survival (19). In 2004, Newman et al. demonstrated the feasibility of continuous, non-invasive cerebral oximetry measurements obtained using NIRS and suggested a possible role for NIRS in evaluating the adequacy of CPR methods (20).

Given the physics of light in the near-infrared spectral region (600–900 nm) within the brain, tissue absorption is mainly determined based on levels of oxygenated and reduced hemoglobin, with smaller contributions from water, lipids, and cytochrome c oxidase. Cerebral saturation is measured using a light source fixed to the head, which transmits infrared and red spectrum light through the skin, skull, connective tissues, and brain. Quantification is then performed using a light detector. The separation between the source and detector is an important parameter of the NIRS system, as it determines the depth of penetration (i.e., ~2–2.5 cm with current systems) (30, 31). Values are thus measured from a “banana-shaped” volume of tissue (32, 33). Furthermore, the intence of near-infrared light is also important because the larger source-detector distance, the deeper the photon reaches inside the brain layer, but the intensity of the detected light decreases more strongly (34, 35).

rSO_2_ values can be affected by various factors, such as extracranial contamination, skin pigmentation (36, 37), and physiological conditions. Changes in physiological conditions may in turn lead to changes in cerebral blood flow or oxygen content. Among the factors known to influence these parameters are cardiac output, acid–base status, major hemorrhage, obstructions of arterial inflow/venous outflow, hemoglobin concentration, hemoglobin saturation, pulmonary function, inspired oxygen concentration, and drug use (e.g., phenylephrine) (38–41).

Currently, there are several commercially available NIRS devices (12, 16, 34, 36, 42–45). These devices differ with regard to the wavelengths and frequencies used, the timing of light transmission, the distance between the light source and detector, and the primary principle of measurement [e.g., Beer-Lambert law (46), spatial-resolved spectroscopy law (47), or time-resolved absorption spectroscopy law (46)]. Thus, the algorithms used to derive hemoglobin saturation from the inputs received also differ for each device. It has been reported that values for rSO_2_ typically range from 55 to 80%, and rSO_2_ <50% or a 20% reduction from the individual baseline is generally considered indicative of the need for intervention (48). However, it is noted that the threshold of the normal range is not clearly defined due to the characteristics of the equipment, and the range of normal values actually varies among the equipment (37). As most clinical NIRS devices assume a venous/arterial distribution in cerebral cortical tissue of ~70/30 or 75/25%, based in part on the results of positron emission tomography studies (39), rSO_2_ values are primarily influenced by cerebral venous oxygen saturation (49). However, previous studies have reported that the venous/arterial distribution of the cerebral cortex varies among individuals (37, 50, 51), suggesting that rSO_2_ values are also variable (50). Previous studies have reported that the absolute values, or different degrees of variability in rSO_2_ due to several factors, vary between NIRS devices under various conditions (36, 37, 45, 52). Given that rSO_2_ values also vary based on physiological conditions, some authors have suggested that relative changes in rSO_2_ from baseline are more appropriate for guiding resuscitative efforts than absolute values (53, 54).

## Search Strategy

To review articles regarding NIRS brain monitoring in patients with CA, we searched PubMed, Web of Science, and Google Scholar for relevant studies. There was no language restriction. We developed a search strategy using the combination of keywords and Medical Subject Heading (MeSH) terms, which were “(Near-infrared spectroscopy [MeSH] OR (regional cerebral oxygen saturation) OR (brain oximetry)) AND ((Heart arrest [MeSH]) OR (cardiac surgery) OR prehospital)” for PubMed and Web of Science, and [“Near-infrared spectroscopy,” “cardiac arrest,” “regional saturation”] for Google Scholar. The main findings of the included studies are summarized in Table 1.

**Table 1:** Summary of main findings in the included studies.

**Author**	**Year**	**Type of cerebral oximeter**	**Type of CA**	**Clinical setting**	**Conclusion**	**Reference**
**EARLIER DETECTION OF RE-ARREST**						
Frisch	2012	InSpectra	OHCA	Prehospital	A decline in rSO_2_ level may correlate with re-arrest.	(24)
Meex	2013	FORE-SIGHT	OHCA		Re-arrest was accompanied with sudden drop in rSO_2_	(55)
Schewe	2014	EQANOX 7600	OHCA	Prehospital-VT	rSO_2_ decreased prior to re-arrest.	(56)
Nomura	2016	HAND ai TOS	OHCA	Prehospital-PEA	Re-arrest PEA was accompanied with sudden drop in rSO_2_	(57)
**ASSESSMENT OF CPR QUALITY**						
Paarmann	2010	INVOX 5100	IHCA	In-hospital	rSO_2_ may be a non-invasive alternative for the assessment of the adequacy of oxygen transport (i.e. CPR efforts).	(58)
Kämäräinen	2012	INVOS 5100c	IHCA	In-hospital	High quality CPR and improving CPR technique was not significantly reflected in rSO_2_ as quantified.	(59)
Meex	2013	FORE-SIGHT/EQUANOX advance	IHCA/OHCA	In-hospital	Decrease in rSO_2_ during interruption of CPR Increase in rSO_2_ due to improved resuscitation efforts	(55)
Schewe	2014	Equanox 7600	OHCA	Prehospital (mechanical CPR)	rSO_2_ during mechanical CPR was higher compared to manual compression	(56)
Parnia	2014	Equanox 7600	IHCA	In-hospital (mechanical CPR)	Mechanical CPR was significantly associated with higher rSO_2_ compared with manual chest compression.	(60)
Ogawa	2015	TOS-OR	OHCA	ER (mechanical CPR)	LDB-CPR significantly increased rSO_2_ value compared with manual CPR.	(61)
**PREDICTION OF ROSC**						
Asim	2014	INVOS 5100c	OHCA	ER	ROSC was established in the patients with rise in rSO_2_.	(62)
Sanfilippo	2015	N/A	IHCA/OHCA		Both initial and average rSO_2_ values were significantly higher in the ROSC group than in the non-ROSC group.	(63)
Cournoyer	2016	N/A	IHCA/OHCA		Mean NIRS value were higher in patients experiencing ROSC than in their respective counterparts.	(53)
Schnaubelt	2018	N/A	IHCA/OHCA		Both mean rSO_2_ and ΔrSO_2_ were higher in the ROSC group than in the non-ROSC group.	(64)
Takegawa	2019	TOS-OR	OHCA	ER	The combination of baseline rSO_2_ with the amount of maximum rise in rSO_2_ over time is better predictor of ROSC.	(65)
**PREDICTION OF FAVORABLE NEUROLOGICAL OUTCOMES**						
Meex	2013	FORE-SIGHT	OHCA	ICU-During TTM	rSO_2_ value was significantly lower in non-survivors compared with survivors at 3 h after induction of TTM.	(66)
Storm	2014	INVOS 5100c	IHCA/OHCA	ICU-During TTM	rSO_2_ within the first 40 h after ROSC is significantly lower in patients with poor neurological outcome.	(67)
Genbrugge	2016	FORE-SIGHT	OHCA	ICU-During TTM	The mean rSO_2_ in the rewarming phase was significantly higher among patients with CPC scores of 1–2.	(68)
Cournoyer	2016	N/A	IHCA/OHCA		Mean NIRS value or combined initial and mean NIRS values were higher in patients with good neurologic outcomes.	(53)
Bougle	2016	INVOS	OHCA	ICU-During TTM	rSO_2_ within 48 h after ICU admission does not allow discriminating patients with good or bad outcome.	(69)
Schnaubelt	2018	N/A	IHCA/OHCA		ROC analysis could not confirm a significant discriminatory power for mean rSO_2_ values.	(64)
Saritas	2018	INVOS	CA	ICU-During TTM	There was no significant correlation between rSO_2_ values and neurologic outcomes.	(70)
Nakatani	2018	INVOS 5100c	OHCA	ER/ICU-During TTM	TTM at 32–34°C effectively decreased all-cause mortality in comatose OHCA patients with rSO_2_ 41–60% on arrival.	(71)
Jakkula	2019	INVOS 5100c	OHCA	ICU	No association between rSO_2_ and NSE at 24, 48, 72 h after OHCA or good neurological outcomes at 6 months.	(72)
Sakurai	2020	INVOS 5100c	OHCA	ICU- During TTM	There was no significant difference in rSO_2_ values between prognosis groups at any time point.	(73)

*CA, Cardiac arrest; OHCA, Out-of-hospital cardiac arrest; IHCA, in-hospital cardiac arrest; rSO_2_, regional cerebral oxygen saturation; ROSC, return of spontaneous circulation; CPR, cardiopulmonary resuscitation; CPC, cerebral performance category; ER, emergency room; ICU, intensive care unit; LDB, load distributing band; NSE, neuron specific enolase; ROC, receiver operating characteristic; TTM, targeted temperature management; N/A, not available*.

## Use of NIRS for Earlier Detection Of Re-Arrest in Prehospital Settings

While ROSC is often successful in pre-hospital settings, many patients subsequently develop circulatory instability and experience re-arrest (i.e., a loss of pulse after sustained ROSC) (74). Since re-arrest before reaching the hospital is among the potential barriers to survival in patients with OHCA (74), early recognition of re-arrest is crucial for ensuring prompt re-activation of resuscitation protocols, including CPR and early defibrillation. Many EMS systems routinely use pulse oximetry measurements; however, pulse oximetry depends on the presence of a peripheral pulse, and the technique is unreliable when used during CA because pulsatile blood flow is inadequate in peripheral tissue beds under such conditions (75, 76). Using a finger pulse oximeter is problematic during CA because any resultant values likely reflect the pulsation of venous blood. Thus, although the presence of a plethysmograph waveform on pulse oximetry is potentially valuable in detecting ROSC, the main purpose of pulse oximetry is to ensure appropriate oxygenation after ROSC, and its use is limited during CPR (76).

In contrast to pulse oximetry, NIRS can measure tissue oxygenation in the absence of pulsatile flow, without the need to interrupt chest compressions (55, 57, 62). Since NIRS values are affected by ambient light (52, 77), some devices cannot be used outside. Nonetheless, rSO_2_ monitoring may aid in the early detection of ROSC and re-arrest in patients with CA (55–57, 62). Meex et al. observed that rSO_2_ values immediately increased after ROSC and that new episodes of ventricular fibrillation were immediately detected as sudden decreases in rSO_2_. These findings suggest that decline in rSO_2_ values can reflect life-threatening situations such as pulseless arrhythmia or severe cerebral hypoperfusion, both of which indicate an urgent need for CPR (55). Additional studies have reported that ROSC is associated with increases in NIRS values, while re-arrest is associated with decreases in NIRS values (56, 57). Notably, these studies showed the decrease in rSO_2_ at the re-arrest episode, which is difficult to find re-arrest without pulse check. It may be useful to be aware of re-arrest immediately without pulse check. Another study reported that NIRS monitoring can aid in assessing perfusion and guiding interventions during transport (78). Some authors have suggested that low NIRS readings highlight the need for additional lifesaving interventions such as fluid resuscitation and/or vasopressors (40, 41, 79). Therefore, NIRS monitoring may enable early recognition of re-arrest, especially in PEA, and poor cerebral circulation during EMS resuscitation protocol. Since vital signs and the results of physical assessments can be influenced by environmental factors (e.g., pre-hospital settings, ambulance transport), further clinical studies are required to determine the value of NIRS in various settings.

## Feasibility of NIRS for the Assessment of CPR Quality

Well-performed CPR has been associated with higher rates of ROSC (80, 81), better cerebral perfusion (82), and improved cerebral oxygenation (83). Several large-scale studies have demonstrated that high-quality CPR improves survival and neurological outcomes among patients with CA (84–86). However, monitoring the adequacy of circulation and cerebral oxygenation during CPR remains challenging. To date, studies investigating the use of NIRS devices to assess the quality of CPR have yielded conflicting results (55, 58, 59).

To assess the quality of CPR, Kämäräinen et al. measured rSO_2_ using an INVOS 5100c device and simultaneously monitored indicators of CPR quality. Compression depth, the rate and release of compressions, and ventilation rate were monitored during CPR with automated real-time audiovisual feedback (59). Data related to the quality of CPR and rSO_2_ were measured at 30-s intervals until ROSC (59). The authors observed that cerebral oxygenation remained low throughout high-quality CPR (59), in contrast to the previous findings that cerebral rSO_2_ decreases due to circulatory arrest during cardiac surgery but increases during CPR (87) or cardiopulmonary bypass (88). However, the rSO_2_ data recorded in this study were unreliable in many cases, as 59% of the 30-s intervals exhibited artifacts that precluded quantification of rSO_2_ (59). In contrast, Meex et al. observed parallel increases in systolic arterial pressure and rSO_2_ during CPR (55), suggesting a positive effect of CPR on these two parameters. In addition, switching CPR providers resulted in a measurable increase in cerebral oxygen saturation. An rSO_2_ decreased to values between 30 and 35% after cessation of CPR. The authors further stated that rSO_2_ monitoring allows for both the continuous estimation of cerebral oxygenation without ROSC and the assessment of CPR efficacy (55). Previous research has indicated that mechanical chest compression, which is thought to provide adequate compression over long periods of time without fatigue or interruption, significantly increases rSO_2_ values in patients with OHCA, in contrast to manual chest compression (61). Although their sample sizes were small, other studies have also noted that mechanical CPR is associated with significantly higher rSO_2_ values than manual CPR (56, 60).

The abovementioned findings indicate that dynamic rSO_2_ monitoring may be more useful than static assessments of rSO_2_ during CPR, as such monitoring can provide quantitative information regarding cardiac output and cerebral perfusion during chest compressions. Application of NIRS for the assessment of CPR quality and oxygen delivery to the brain may thus help to improve clinical outcomes following CA. Further studies are required to determine how NIRS monitoring can be integrated into standardized CPR protocols.

## Prediction of ROSC and Favorable Neurological Outcomes

International guidelines recommend end-tidal CO_2_ (ETCO_2_) monitoring for the assessment of CPR quality, noting that a sudden increase in ETCO_2_ is likely to represent an early indicator of ROSC (89). The potential value of ETCO_2_ for optimizing resuscitation efforts is discussed elsewhere (89). However, ETCO_2_ readings are influenced by mechanical ventilation settings, the tidal volume of ventilation, many drugs administered during resuscitation, and by different lung pathologies. In addition, ETCO_2_ monitoring does not provide data related to cerebral circulation. Thus, ETCO_2_ monitoring is distinctly different from NIRS monitoring. In a recent prospective study by Engle et al., ETCO_2_ assessments and cerebral oximetry were performed simultaneously during CPR in the ED (90). The authors observed that both ETCO_2_ and rSO_2_ were good predictors of ROSC. However, logistic regression analysis of the simultaneously collected data revealed that rSO_2_ was superior to ETCO_2_ in predicting ROSC (90).

A 2015 systematic review and meta-analysis reported that both initial and average rSO_2_ values were significantly higher in the ROSC group than in the non-ROSC group (63). An extensive 2016 meta-analysis including 20 studies demonstrated that mean NIRS values were higher in patients experiencing ROSC, surviving to discharge, and surviving with good neurologic outcomes than in their respective counterparts (53). The authors further reported that combined initial and mean NIRS values were higher in patients who survived to discharge and in those who experienced good neurological outcomes than in their counterparts (53). In the most recent systematic review and meta-analysis of 26 studies, Schnaubelt et al. demonstrated that both mean rSO_2_ and ΔrSO_2_ (i.e., the difference between the initial value and the value at ROSC, or the difference between the initial value and the value at the end of CPR) were higher in the ROSC group than in the non-ROSC group (64). ROSC was not observed when mean rSO_2_ remained <26%. An rSO_2_ threshold of 36% predicted ROSC with a sensitivity of 67% and specificity of 69%, while a ΔrSO_2_ of 7% predicted ROSC with a sensitivity of 100% and a specificity of 86% [area under the curve (AUC) = 0.733 and 0.893, respectively] (64).

However, given that baseline values vary among patients (54), comparisons of static values obtained using different devices may be methodologically problematic (91, 92). Importantly, all studies in these meta-analyses focused on averages obtained from static values, rather than on changes in NIRS readings within the same patient. Thus, it is difficult to determine the absolute cut-off value for discontinuing CPR based on the currently available data, as some patients experienced ROSC even with rSO_2_ values lower than the suggested cut-off values. Furthermore, some authors have suggested that dynamic assessments of rSO_2_ obtained throughout resuscitation are more appropriate than static assessments for evaluating outcomes in patients with CA (22). In a single-center retrospective study, Takegawa et al. evaluated the association between the probability of ROSC and the degree of rSO_2_ increase during CPR among 90 patients with OHCA, 35 (38.9%) of whom achieved ROSC (65). Receiver operating characteristic curve (ROC) analysis revealed that the amount of maximum rise in rSO_2_ value (i.e., the difference between maximum and baseline values) over a 16-min measurement period yielded an AUC of 0.75 for differentiating between the ROSC and non-ROSC groups. In addition, the best AUC value was achieved by the combination of the amount of maximum rise and baseline rSO_2_, rather than by the amount of maximum rise alone (AUC = 0.91) (65). The authors suggested that discontinuation of CPR may be indicated in patients with low initial values who do not exhibit an appropriate increase in rSO_2_, resulting in a low mean value. Taken together, the available data suggest that average rSO_2_ and ΔrSO_2_ values during CPR may aid in determining the likelihood of achieving ROSC in patients with CA. Given that it is difficult to measure mean rSO_2_ during on-going CPR in real-world settings, it is reasonable to focus on the combination of baseline rSO_2_ and ΔrSO_2_ during CPR. Further large-scale, prospective, multicenter studies are required to assess the ability of ΔrSO_2_ to predict ROSC.

Previous studies have reported good neurologic outcomes following CA in patients with both high initial rSO_2_ values and high mean rSO_2_ values (53). In the most recent meta-analysis, the calculated averaged mean rSO_2_ values were higher in patients with favorable neurological outcomes (Glasgow–Pittsburgh Cerebral Performance Category [CPC]: 1 or 2) than in those with poor neurological outcomes (rSO_2_: 47 vs. 38%, *P* = 0.018) (64). CPC scores of 1 or 2 were not observed in patients with mean rSO_2_ values ≤ 30 ± 17%. Mean rSO_2_ values in patients with favorable neurological outcomes were significantly above 30%. However, ROC analysis for neurological outcomes could not confirm a significant discriminatory power for mean rSO_2_ values (AUC = 0.770, *P* = 0.098), likely due to the small sample size (64). The authors concluded that mean rSO_2_ and ΔrSO_2_ values have good predictive value for ROSC but not for favorable neurological outcomes (64). Moreover, in a *post hoc* analysis of a randomized clinical trial, Jakkula et al. observed no association between cerebral rSO_2_ (median rSO_2_ during the first 36 h) and concentrations of neuron-specific enolase (a marker of neurological injury) at 24, 48, and 72 h after OHCA or good neurological outcomes at 6 months (72). Despite the promising trends suggested by the available evidence, clear cut-off values of rSO_2_ for predicting favorable outcomes after CA are yet to be established.

## Use of NIRS During TTM

Given that induction of hypothermia affects cerebral oxygen metabolism and changes the balance between oxygen supply and demand (93), several studies have examined the role of NIRS monitoring during TTM (66–71, 73). Although some small-scale studies have applied NIRS monitoring during and after TTM in patients with PCAS, meta-analyses or systematic reviews on NIRS monitoring during TTM have been extremely limited. Meex et al. evaluated serial changes in rSO_2_ during TTM in 28 patients with OHCA who underwent hypothermia at 33°C for 24 h after ROSC (66). Values for rSO_2_ decreased significantly within 3 h after the onset of TTM, indicating that the balance between oxygen supply and demand may have been adversely affected. After 3 h, rSO_2_ gradually increased again even during hypothermia, increasing further during the 12-h rewarming period. Although there was no significant difference in rSO_2_ between the survival and non-survival groups, the decrease in rSO_2_ observed during the early stages of hypothermia was significantly greater in the non-survival group than in the survival group (66). Other studies have also reported a general trend that rSO_2_ values decrease after the onset of hypothermia, increasing during and after rewarming (68, 70). These results were contrary to the expectation that rSO_2_ values should increase due to reductions in brain metabolism/oxygen consumption and the effects of hypothermic conditions on the affinity of hemoglobin for oxygen (66). Therefore, the contrary results were likely due to increases in cerebrovascular resistance and decreases in cerebral blood flow.

There are several possible explanations for decreases in rSO_2_ during the early phase of TTM. Some investigators have suggested that cerebral blood flow and rSO_2_ are influenced by cardiac output, use of α-adrenergic vasoconstrictor agents (40, 41, 94), use of anesthetic agents, or other confounding factors. Some studies have also reported that rSO_2_ values during TTM are associated with neurological prognosis (67, 68, 71). Storm et al. evaluated the association between rSO_2_ values and neurological outcomes at hospital discharge and 6 months later in 60 patients with in-hospital cardiac arrest and OHCA. Continuous measurements of cerebral rSO_2_ were obtained for 40 h (i.e., from the onset of hypothermia to rewarming). Median rSO_2_ values at all time points (i.e., at the start of measurement; upon reaching 33°C; and at 4, 12, 24, and 40 h) were persistently higher in patients with CPC scores of 1–2 than in patients with CPC scores of 3–5 (median rSO_2_: 68 vs. 58%, *P* < 0.01) (67). However, rSO_2_ levels largely overlapped between outcome groups, suggesting that the potential of rSO_2_ to aid in predicting outcomes is limited (67). Genbrugge et al. reported that the mean rSO_2_ value during rewarming following hypothermia was significantly higher among patients with CPC scores of 1–2 than among those with CPC scores of 3–5 (70 ± 1 vs. 68 ± 1%, *P* = 0.046) (68). However, they also mentioned that significant differences in rSO_2_ in their study were unlikely to be clinically meaningful given that such data are not available at the bedside. Moreover, given the small sample size of the study, their data cannot be used to determine cutoff rSO_2_ values for predicting outcomes (68). In contrast, other studies have reported no significant differences in rSO_2_ values between prognosis groups, even when changes in rSO_2_ values over time were investigated (69, 70, 73).

Given the available evidence, further studies are required to validate the efficacy of rSO_2_ values during the early stages of TTM in predicting outcomes in patients with PCAS. Stratifying patients according to severity based on rSO_2_ values (71) may aid in distinguishing which patients would benefit from hypothermia. Further large-scale, prospective, multicenter studies are required to elucidate the potential of rSO_2_ during TTM for predicting neurological outcomes following CA.

## Conclusion

**Figure 1:**
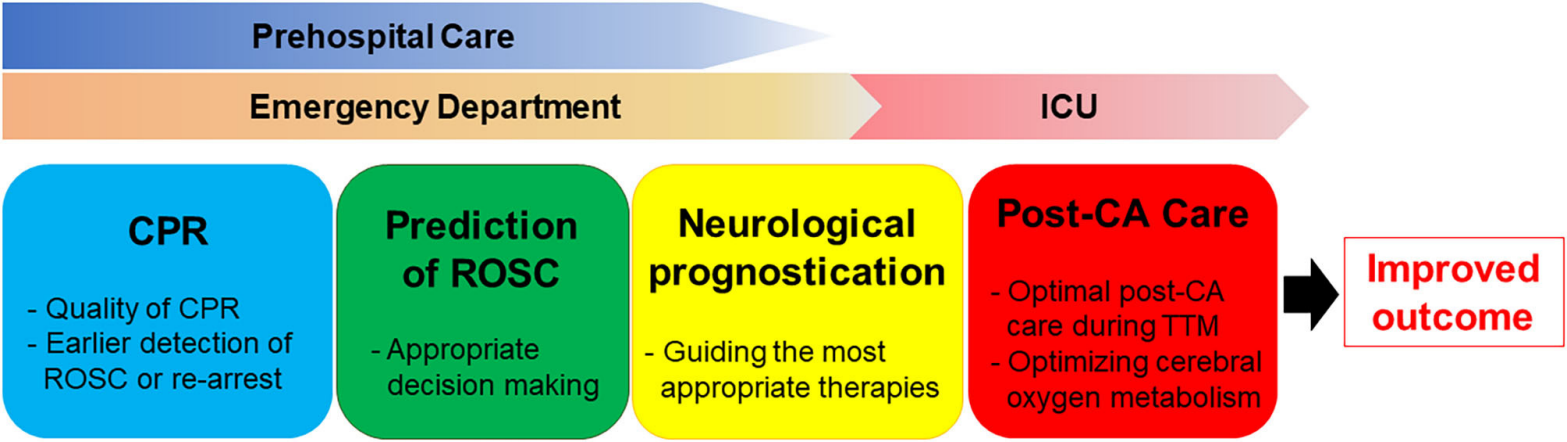
The clinical relevance of NIRS brain monitoring as a predictive tool in both pre-hospital and in-hospital settings.

In the present review, we summarized the development, evolution, and potential application of near-infrared spectroscopy (NIRS) in adults with CA, highlighting the clinical relevance of NIRS brain monitoring as a predictive tool in both pre-hospital and in-hospital settings (Figure 1). To date, no studies have established acceptable rSO_2_ cut-off values for differentiating patient groups based on ROSC status and neurological outcome. Furthermore, the extent of decrease in rSO_2_ from baseline that constitutes an abnormal finding in patients with CA remains to be determined. Additional studies are required to determine the point at which resuscitation efforts can be considered futile. Nonetheless, the available evidence indicates that rSO_2_ may aid not only in predicting outcomes among patients with CA, but also in optimizing CPR strategies and guiding neuroprotective interventions. Further large-scale randomized controlled trials are required to evaluate the impact of NIRS monitoring on survival and neurologic recovery. Moreover, additional studies should evaluate NIRS-guided resuscitative strategies, using improvements in NIRS values to optimize resuscitation efforts, post-resuscitation care, and patient outcomes.

## Author Contributions

RT and KH: concept, design, and drafting manuscript. DR, TL, SM, MO, TS, and LB: critical revision of the manuscript for important intellectual content. All authors: have read and approved the manuscript.

## Conflict of Interest

The authors declare that the research was conducted in the absence of any commercial or financial relationships that could be construed as a potential conflict of interest.

## Abbreviations

OHCA: out-of-hospital cardiac arrest
EMS: emergency medical services
TTM: targeted temperature management
PCAS: post-cardiac arrest syndrome
rSO_2_: regional cerebral oxygen saturation
NIRS: near-infrared spectroscopy
ICU: intensive care unit
CPR: cardiopulmonary resuscitation
ROSC: return of spontaneous circulation
ED: emergency department
ETCO_2_: end-tidal CO_2_
AUC: area under the curve
ROC: receiver operating characteristic curve
CPC: Glasgow–Pittsburgh Cerebral Performance Category.
