# Supplementary material for: Near-Infrared Spectroscopy Assessments of Regional Cerebral Oxygen Saturation for the Prediction of Clinical Outcomes in Patients With Cardiac Arrest: A Review of Clinical Impact, Evolution, and Future Directions
Source: Front Med (Lausanne). 2020 Oct 29;7:587930. doi: 10.3389/fmed.2020.587930 (PMC7673454; doi:10.3389/fmed.2020.587930)
Supplement: Supplementary file 1 [file Data_Sheet_1.PDF]

Author	Year	Type of cerebral oximeter	Type of CA	Clinical setting	Conclusion	Ref.
**Earlier detection of re-arrest**						
Frisch	2012	InSpectra	OHCA	Prehospital	A decline in rSO_2 level may correlate with re-arrest.	24
Meex	2013	FORE-SIGHT	OHCA		Re-arrest was accompanied with sudden drop in rSO_2	58
Schewe	2014	EQANOX 7600	OHCA	Prehospital- VF	rSO_2 decreased prior to re-arrest.	62
Nomura	2016	HAND ai TOS	OHCA	Prehospital- PEA	Re-arrest PEA was accompanied with sudden drop in rSO_2	60
**Assessment of CPR quality**						
Paarmann	2010	INVOX 5100	IHCA	In-hospital	rSO_2 may be a non-invasive alternative for the assessment of the adequacy of oxygen transport (i.e. CPR efforts).	72
Kämäräinen	2012	INVOS 5100c	IHCA	In-hospital	High quality CPR and improving CPR technique was not significantly reflected in rSO_2 as quantified.	73
Meex	2013	FORE-SIGHT/ EQUANOX Advance	IHCA/OHCA	In-hospital	Decrease in rSO_2 during interruption of CPR<br>Increase in rSO_2 due to improved resuscitation efforts	58
Schewe	2014	Equanox 7600	OHCA	Prehospital (mechanical CPR)	rSO_2 during mechanical CPR was higher compared to manual compression	62
Parnia	2014	Equanox 7600	IHCA	In-hospital (mechanical CPR)	Mechanical CPR was significantly associated with higher rSO_2 compared with manual chest compression.	77
Ogawa	2015	TOS-OR	OHCA	ER (mechanical CPR)	LDB-CPR significantly increased rSO_2 value compared with manual CPR.	76
**Prediction of ROSC**						
Asim	2014	INVOS5100C	OHCA	ER	ROSC was established in the patients with rise in rSO_2.	59
Sanfilippo	2015	N/A	IHCA/OHCA		Both initial and average rSO_2 values were significantly higher in the ROSC group than in the non-ROSC group.	80
Cournoyer	2016	N/A	IHCA/OHCA		Mean NIRS value were higher in patients experiencing ROSC than in their respective counterparts.	53
Schnaubelt	2018	N/A	IHCA/OHCA		Both mean rSO_2 and ΔrSO_2 were higher in the ROSC group than in the non-ROSC group.	81

Takegawa	2019	TOS-OR	OHCA	ER	The combination of baseline rSO_2 with the amount of maximum rise in rSO_2 over time is better predictor of ROSC.	84
**Prediction of favorable neurological outcomes**						
Meex	2013	FORE-SIGHT	OHCA	ICU -During TTM	rSO_2 value was significantly lower in non-survivors compared with survivors at 3h after induction of TTM.	87
Storm	2014	INVOS 5100c	IHCA/OHCA	ICU -During TTM	rSO_2 within the first 40 hours after ROSC is significantly lower in patients with poor neurological outcome.	90
Genbrugge	2016	FORE-SIGHT	OHCA	ICU -During TTM	The mean rSO_2 in the rewarming phase was significantly higher among patients with CPC scores of 1-2.	88
Cournoyer	2016	N/A	IHCA/OHCA		Mean NIRS value or combined initial and mean NIRS values were higher in patients with good neurologic outcomes.	53
Bougle	2016	INVOS	OHCA	ICU -During TTM	rSO_2 within 48 hours after ICU admission does not allow discriminating patients with good or bad outcome.	93
Schnaubelt	2018	N/A	IHCA/OHCA		ROC analysis could not confirm a significant discriminatory power for mean rSO_2 values.	81
Saritas	2018	INVOS	CA	ICU -During TTM	There was no significant correlation between rSO_2 values and neurologic outcomes.	89
Nakatani	2018	INVOS 5100c	OHCA	ER/ICU -During TTM	TTM at 32–34 ℃ effectively decreased all-cause mortality in comatose OHCA patients with rSO_2 41–60% on arrival.	91
Jakkula	2019	INVOS 5100c	OHCA	ICU	No association between rSO_2 and NSE at 24, 48, 72 hours after OHCA or good neurological outcomes at 6 months.	85
Sakurai	2020	INVOS 5100c	OHCA	ICU- During TTM	There was no significant difference in rSO_2 values between prognosis groups at any time point.	92

Table. Summary of main findings in the included studies.
CA; Cardiac arrest, OHCA; Out-of-hospital cardiac arrest, IHCA: in-hospital cardiac arrest, rSO_2; regional cerebral oxygen saturation, ROSC; return of spontaneous circulation, CPR; cardiopulmonary resuscitation, CPC; cerebral performance category, ER; emergency room, ICU; intensive care unit, LDB; load distributing band, NSE; neuron specific enolase, ROC; receiver operating characteristic, TTM; targeted temperature management, N/A; not available.
